# Changing landscapes in cellular oncology

**DOI:** 10.1007/s13402-012-0090-8

**Published:** 2012-07-11

**Authors:** Ad Geurts van Kessel

**Affiliations:** Department of Genetics, Radboud University Nijmegen Medical Centre, PO Box 9101, 6500 HB Nijmegen, The Netherlands

In his editorial in the February 2011 issue of Cellular Oncology Gerrit Meijer pointed out that with the onset of the second decade of this century ‘many of us expect to witness the full maturation of omics being turned into diagnostic and therapeutic applications that will improve clinical outcome in many diseases, including oncology’. It has indeed been advocated for some time that one day we may stratify patients using in-depth omics analyses. Thanks to the diminishing costs and increasing speed of high-throughput technologies this era has now begun, and tabletop instruments with the ability to genotype and analyze patient samples in less than 45 min are well underway. In the recent past, several laboratories around the world have teamed up in the International Cancer Genome Consortium (ICGC) following the launch of comprehensive cancer genome projects in the UK and the USA, and the sequences of cancer genomes are being published now at an increasingly rapid pace. Based on these developments, a growing list of companies and hospitals have begun to employ next generation sequencing technologies to identify relevant mutations across genomes for diagnostic and prognostic purposes, and to apply them to potentially targeted therapies. In spite of these exciting developments, however, several questions remain, including uncertainties about whether our understanding of the cancer genome is already advanced enough to make a comprehensive use of these technologies effective. Indeed, the biological relevance of many sequence variants is, as yet, poorly understood. To meet this interpretational challenge, it will be essential to combine the expertise of geneticists, pathologists, oncologists and bio-informaticians, and to validate preclinical results in well-characterized population-based cohorts and randomized clinical trials. The ultimate clinical adoption of omics technologies will require broad collaborations across researchers, industries, health care workers and policy makers.

Over the last several years Cellular Oncology has played an increasingly prominent role in covering topics related to the pathogenesis of cancer, the development of new technologies and its application to early diagnosis and optimized patient stratification for personalized treatment. Gerrit Meijer has significantly contributed to the strong position of the journal in these fields of oncology and we owe him and his editorial team much gratitude for that. During the combined ISCO-EWCMST meeting at La Palma de Mallorca, Spain earlier this year (see report in this issue) he has handed over this task to a new team that, together with Springer and ISCO, has taken on the challenge to move the field of cellular oncology to a next level and to contribute to the prospect that lies ahead of us, i.e., the maturation of omics technologies into comprehensive diagnostic and therapeutic applications. In order to engage in this challenge, the new editorial team strongly encourages scientists from various disciplines to contribute to the journal, not only their original research work, but also state-of-the-art reviews to update peers and all those interested on the rapidly changing landscapes in cellular oncology.

